# Exposure to particulate matter, prenatal depressive symptoms and HPA axis dysregulation

**DOI:** 10.1016/j.heliyon.2021.e07166

**Published:** 2021-05-28

**Authors:** Nina E. Ahlers, Sandra J. Weiss

**Affiliations:** Department of Community Health Systems, University of California, San Francisco, CA, USA

**Keywords:** Air pollution, Particulate matter, Cortisol, Prenatal depression, HPA dysregulation

## Abstract

**Background:**

The prevalence of depression during pregnancy is on the rise, affecting women's well-being and their children's health outcomes. Preliminary studies suggest that exposure to air pollution during pregnancy may play a role in development of depressive symptoms. In addition, pollution has been linked to dysregulation of the hypothalamic-pituitary-adrenal (HPA) axis, our brain's primary stress response system. The purpose of this study was to examine the association of air pollution exposure during pregnancy to prenatal depressive symptoms. We also evaluated whether cortisol, the hormonal endpoint of HPA activation, mediated the relationship between exposure to pollution and prenatal depression.

**Methods:**

Women were recruited in obstetric clinics during their third trimester of pregnancy. They completed the Patient Health Questionnaire-9 to assess depression and provided salivary samples at 4 times during the day for 2 days. Four measures of cortisol were calculated from salivary assays: average cortisol level, cortisol awakening response (CAR), diurnal cortisol slope (DCS), and area under the curve (AUC_G_). We acquired data on particulate matter with a diameter of _2.5_ μm (PM_2.5_) or less within each woman's residential area from public records of the air quality control district. Structural equation modeling was used to analyze the aims.

**Results:**

Increased prenatal exposure to PM_2.5_ across pregnancy was associated with more severe depressive symptoms during the 3rd trimester (β = 0.14, p = 0.02). Greater PM_2.5_ exposure also had significant relationships with both higher cortisol AUC_G_ (β = 15.933, p = 0.005) and average cortisol levels (β = 0.018, p = 0.023) among women. However, no cortisol parameter appeared to mediate the relationship between PM_2.5_ exposure and depressive symptoms.

**Conclusions:**

Findings suggest pregnancy may be a critical window of sensitivity to PM_2.5_ exposure that escalates depression risk and induces activation of the HPA axis, evidenced in greater overall cortisol concentration. Further research is needed to identify mechanisms underlying the effects of particulate matter, especially potential methylation of glucocorticoid or serotonin transporter genes that may elicit changes in both depression and the stress response system. In addition, assessment of depression appears warranted for pregnant women in regions known for high pollution.

## 1 Introduction

Over the last 2 decades, studies indicate that the prevalence of depression during pregnancy is on the rise, with rates moving from around 8 to 12% at the start of this century (Bennett et al., 2004) to 23–25% more recently (Fekadu Dadi et al., 2020; Pearson et al., 2018). Reported rates of prenatal depression reach a staggering 34–38% in certain high-risk groups (Fekadu Dadi et al., 2020; Mochache et al., 2018). Prenatal depression is a significant risk factor for postnatal depression in both developed and developing countries (Norhayati et al., 2015) and is linked to obstetric problems and adverse birth outcomes (Marcus et al., 2009; Räisänen et al., 2014). While the majority of incident depressive symptoms occur during pregnancy rather than afterward (Wilcox et al., 2020), most studies have focused on the detection and treatment of *postpartum* mental health problems (Gavin et al., 2005).

In order to determine best practices in screening and potential targets for treatment of prenatal depression, it is essential to better understand its underlying etiology. Recent studies suggest that exposure to air pollution during pregnancy may play a role in development of depressive symptoms and postpartum depression (Niedzwiecki et al., 2020; Sheffield et al., 2018). One study also has shown an association of exposure to particulate matter from mid to late pregnancy with higher levels of psychological distress during pregnancy (Lin et al., 2017), although they did not find an association specifically with depression. To our knowledge, the association between exposure to air pollution in different trimesters of pregnancy and prenatal depression has not been examined.

In addition, the mechanisms responsible for any relationship between exposure to air pollution and depression remain unclear. One of the most widely accepted pathophysiological factors associated with depression is a dysfunctional hypothalamic-pituitary-adrenal (HPA) axis, which mediates the endocrine stress response in humans (Gonul et al., 2017). It has been proposed that pollution may induce dysregulation of the HPA axis which in turn mediates depressive symptoms and other CNS effects (Thomson et al., 2019). Thomson (2014; Thomson et al., 2013), using a rodent model, found that hormones of the HPA axis and their metabolites were increased immediately after pollutant inhalation. In fact, expression of known glucocorticoid-responsive genes was transiently increased in most organs, consistent with glucocorticoid activity. Thomson et al. (2019) found that exposure to particulate pollutants can rapidly activate the HPA axis to release glucocorticoid stress hormones. Studies in humans also have linked increased air pollution exposure to elevated serum levels of HPA axis hormones (Niu et al., 2018; Li et al., 2017a, Li et al., 2017b), implicating air pollutants as environmental stressors that contribute to activation of the HPA axis (and cortisol secretion). Glucocorticoids in the HPA axis regulate a variety of processes, exerting strong effects on the central nervous system, including changes in mood. In particular, it is well documented that long-term activation of the stress system and HPA axis disturbance are associated with risk of depression (Nandam et al., 2020; Young et al., 2016; Chrousos, 2009). Further, depression has been linked to variation in pollutant levels (Lim et al., 2012; Szyszkowicz et al., 2009). These convergent findings suggest that HPA axis dysregulation could play a mediating role in bringing about prenatal depression after exposure to air pollution. Pregnant women could be particularly susceptible to toxic air exposure due to the dynamic physiology during pregnancy, including changes in cortisol and other related hormones (Li et al., 2017a, Li et al., 2017b; Lin et al., 2017; Sun et al., 2015; Entringer et al., 2010; Weetman, 2010). To our knowledge, no study has examined the mediating role of the HPA axis (as measured via downstream cortisol) in air pollution-induced depression among pregnant women. In this research, we sought to address this gap in knowledge.

Our research aims were: 1) to examine the association between air pollution exposure during pregnancy and prenatal depression during the 3rd trimester, and 2) to determine if specific cortisol parameters mediate the relationship between air pollution exposure and maternal depression.

## 2 Material and methods

### 2.1 Sample and procedures

Participants included a group of women from a larger cohort of 179 participants enrolled in a NIH-funded study (RO1 HD081188-05, S. Weiss PI). Women in the larger cohort were recruited either during their 3^rd^ trimester of pregnancy (n = 83) or shortly after delivery (n = 96) in obstetric clinics and community health centers between 2015 and 2019. English and Spanish speaking women were eligible to participate if they were ≥18 years old and ≥28 week's gestation. Exclusion criteria included being too psychologically or physically ill to participate, having a cognitive impairment, having an adrenal or endocrine disorder, or using a prescribed steroid medication (oral, inhalation, or topical). For the full cohort of 179 women, the mean age was 33 years. About 46% had 2 years of college or less and approximately 42% reported a household income of less than $60,000. 50% of the women were White/European American, 18% Black/African American, 10% Asian American, and 27% of Hispanic/Latina ethnicity. Only data from the women recruited during pregnancy were used in this study. As shown in Table 1, the characteristics of women in this study sample were very similar to those of the larger cohort. Mean age was identical and both income and educational levels were very similar. While the percent of White women was approximately the same, this sample had more Black women (32% versus 18%), fewer Asian Women (6% versus 10%), and fewer Hispanic women (14% versus 27%) than the larger cohort.

**Table 1:** Characteristics of women in the sample on sociodemographics and key study variables.

Variable	Unit or Category	Mean (min-max) or N (%)
Maternal age	Years	33 (21–44)

Maternal education		
	Elementary school	3 (6%)
	High school or GED	8 (16%)
	Some college or 2 year college	13 (26%)
	4 year college degree	9 (18%)
	Master's degree Professional degree (e.g. MD, PhD)	9 (18%) 8 (16%)

Household income		
	Less than $15,000	11 (22%)
	$15,000-$30,999	11 (22%)
	$31,000-$50,999	3 (6%)
	$51,000-$100,999	-
	$101,000-$149,999	8 (16%)
	$150,000+	17 (34%)
Stressors	Number of Events	7.3 (0–39)
Depression	Continuous Score	6.4 (1–20)

PM_2.5_ exposure		
1^st^ trimester exposure	μg/m^3^	8.8 (4.2–20.7)
2^nd^ trimester exposure	μg/m^3^	8.3 (4.2–21.1)
3^rd^ trimester exposure	μg/m^3^	7.4 (5–10.6)
Average pregnancy exposure	μg/m^3^	8.1 (5.8–12.6)

Cortisol parameters^a^		
2 day average	μg/dl	0.25 (0.03–0.55)
CAR	μg/dl	0.02 (-0.3-0.29)
AUC_G_	μg/dl	184.66 (32.18–337.95)
DCS	μg/dl	0.24 (-0.28-0.75)

a 2 day average (average cortisol levels across 2 days), CAR (cortisol awakening response), AUC_G_ (area under the curve), DCS (diurnal cortisol slope).

Women were contacted about the study at some point between 28 weeks of gestation until birth. If they expressed interest in participation, they were provided informed consent. Self-reported socioeconomic information was acquired at enrollment. Women completed depression questionnaires and provided salivary cortisol samples 4 times during the day over a 2 day period shortly after recruitment (details are described below). We acquired data on particulate matter (PM_2.5_) within each woman's residential area from public records of the air quality control district, identifying levels present during each trimester of the woman's pregnancy. The study was approved by the University's Institutional Review Board for Human Research Protection.

### 2.2 Air pollutant (PM_2.5_) exposure during pregnancy

We chose particulate matter with a diameter of or less than 2.5 μm (PM_2.5_) as the main air pollutant of interest because of its most consistent association to depression and hormonal dysregulation in previous research. The Bay Area Air Quality Management District (BAAQMD) (2019) provided 24-h average PM_2.5_ data from 33 air monitoring stations across 9 Bay Area counties with fixed locations based on knowledge of population density and local wind patterns, with the final site selection determined after analyzing preliminary air quality measurements collected from field studies, temporary monitoring studies, and mobile monitoring data. Our participants resided within 4 counties and data from 6 monitors were used. Only one county had more than one monitor (n = 3); however, most of our participants resided in a county with only one monitor (88%). Pregnancy air pollution measures were estimated for each participant according to the nearest monitoring station to maternal residence at time of enrollment. Maternal residence (defined as the primary residence of the entire prenatal period), was geocoded with Google Earth. The average distance between maternal residence and the nearest PM_2.5_ monitoring station was 2.39 miles and ranged between 0.28 to 5.63 miles. Our approach to measurement is supported by Kim et al. (2005) who suggest that central fixed-site measurements of PM_2.5_ can be treated as a proxy measure for personal exposure to PM_2.5_ within a 15.5 mile radius. We calculated four air pollution measures for the analyses, including the average PM_2.5_ exposure over pregnancy and estimates for each clinically defined trimester (1st trimester: 1–13 weeks, 2nd trimester: 14–27 weeks, 3rd trimester: 28 weeks-delivery).

### 2.3 Self-reported depression scores

The 9 item Patient Health Questionnaire (PHQ-9) was administered in English or Spanish and completed by all pregnant women during their third trimester to assess depression. The measure has shown internal consistency and test-retest reliability as well as criterion, discriminant, and construct validity (Kroenke et al., 2001; Kroenke et al., 2010). Respondents rated how frequently they had experienced symptoms over the past two weeks on a scale ranging from 0 = “not at all” to 3 = “nearly every day.” Total scores range from 0 to 27 points, with diagnostic ranges that include minimal symptoms of depression (1–4), mild (5–9), moderate (10–14), moderately severe (15–19), and severe (20+). A score of >10 has been demonstrated to have both a sensitivity and specificity of 88% for a diagnosis of major depression (Kroenk et al., 2001).

### 2.4 Cortisol Samples

Women were given saliva collection kits at recruitment or through the mail, along with verbal and written instructions on how to collect the saliva samples at home. The mean week of gestation for salivary collection was 31 weeks (28–37wks). Using the passive drool method, they provided 1 ml of saliva into a cryovial 4 times each day for 2 days. Samples were collected after waking (1^st^), 45 min after waking (2^nd^), at 4pm (3^rd^), and right before going to bed (4^th^). After collection, mothers were instructed to log each sample time event and store their samples in their household freezer before the research assistant's pre-scheduled pickup date which was no more than 2 weeks after kit delivery. The research assistant subsequently transported the samples to a laboratory facility for -80 °C storage until sent for processing to Salimetrics for cortisol assay within one year of collection (Salimetrics, State College, PA, www.salimetrics.com). Our sample handling and storage adherence is in compliance with the Salimetrics collection and storage protocol (Saliva Collection Handbook. Salimetrics, LLC). Only completed samples with recorded time adherence were included in the analysis. Time adherence includes the 1st sample taken no more than 15 min after waking, the second sample taken between 30 and 45 min after the first sample, and the 3rd sample taken between 3pm to 6pm. In addition, the Medication Event Monitoring System (MEMS caps; Haberer, 2013) was used with a subsample of the entire cohort to assess their adherence in dates and times of saliva collection in comparison to matched controls. Means for adherence were similar between the groups, with data suggesting a high degree of compliance across participants. Samples were assayed in duplicate to determine cortisol levels using a highly sensitive enzyme immunoassay (ELISA). The test used 25 μl of saliva per determination and has a lower limit of sensitivity of 0.007 μg/dl, standard curve range from 0.012 μg/dl to 3.0 μg/dl, an average intra-assay coefficient of variation of 4.6%, and an average inter-assay coefficient of variation of 5.9%.

Four measures of cortisol were calculated from salivary assays: mean cortisol level, cortisol awakening response (CAR), diurnal cortisol slope (DCS), and area under the curve (AUC_G_). The mean cortisol level was derived by averaging the total cortisol concentration of the 8 samples across the 2 days of sampling. The two day mean for each of the four time points was used to calculate the CAR, DCS, and AUC scores. Only two participants had not provided the full 8 samples (≥6 of 8) but had at least completed one full day of sample collection. For these women, the cortisol values for their single complete day were used in calculating cortisol measures. The CAR score was the difference between cortisol level from wake time to 45 min following wake time (Alder et al., 2011), measuring the expected cortisol surge that occurs in the morning. The DCS was calculated as the linear degree of change in cortisol levels across the day from initial waking to evening, excluding the second sampling (morning awakening response). AUC_G_ measured total cortisol secretion across the day, considering the difference of single measurements from one another and the time between each sampling period (Khoury et al., 2015). The trapezoidal formula developed by Pruessner et al. (2003) was used to calculate AUC_G_.

### 2.5 Covariates

Major confounders potentially linked to depression and air pollution exposure were considered for inclusion in testing of the aims. Covariates of age, income bracket, and education level were collected by the sociodemographic questionnaire. Scores for stressors experienced by the women during the 3^rd^ trimester were also included in the analyses. Stressors were measured by *The Crisis in Family Systems (CRISYS) Questionnaire,* administered in English or Spanish (Berry et al. 2001, 2006). Respondents were asked to identify whether they had experienced any of 64 major life events in the past 6 months within 11 domains: financial, legal, career, relationship, home safety, neighborhood safety, medical issues (self and others), home, prejudice, and authority. Adverse life events were summed for each woman, with higher scores indicating greater exposure to stressors in life.

### 2.6 Analysis

**Figure 1:**
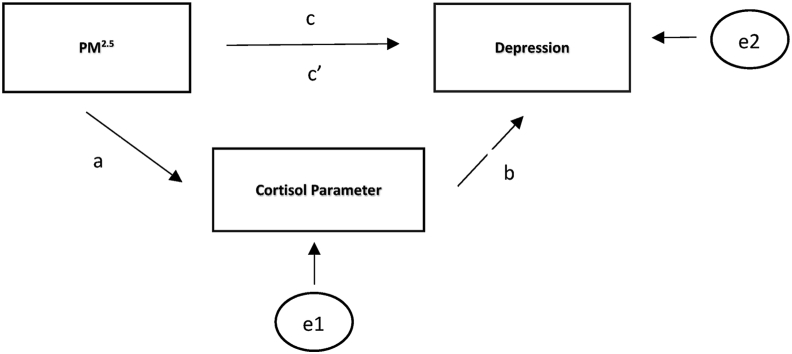
General mediation model and conceptual framework for cortisol's hypothesized mediating role in the relationship between air pollution and depression.

Descriptive statistics were calculated to characterize the sample. Variables were examined for linearity and normality. One participant was excluded from the statistical analysis because of cortisol values that exceeded an expected, normal range. Multiple linear regression procedures were used to examine the association between air pollution exposure during pregnancy and prenatal depression during the 3rd trimester. In separate models, scores for depressive symptoms were regressed on PM_2.5_ exposure for each trimester and for average PM_2.5_ exposure across pregnancy. Covariates were added one by one into the models based on their effect values (see Table 2). As illustrated in Figure 1, we used Structural Equation Modeling (SEM) to examine Aim 2. With SEM, we were able to fit a single model while estimating error variance parameters (e1, e2) for both independent and mediating variables respectively, and permitting the estimation of latent variables from observed variables (Woody, 2011). The path coefficients, a, b, and c represent the effects between the two corresponding variables for each path. The direct effect is represented by c’ (pertaining to the unmediated effect of PM_2.5_ on depression) and the indirect effect is the effect of PM_2.5_ on depression that is mediated by each cortisol parameter (the product of *a* times *b* (a∗b)). The total effect is the sum of the direct and indirect effects (c’ + a∗b).

**Table 2:** Bivariate correlations between key study variables and covariates.

	Depression	1^st^ Trimester PM_2.5_	2^nd^ Trimester PM_2.5_	3^rd^ Trimester PM_2.5_	Average Pregnancy PM_2.5_	Mean Cortisol	CAR	AUC_G_	DCS	Age	Education	Income
Depression	1											
1^st^trimester PM_2.5_	**0.296**∗	1										
2^nd^trimester PM_2.5_	.0152	0.150	1									
3^rd^trimester PM_2.5_	0.108	-0.219	-0.182	1								
Average PM_2.5_	**0.343**∗	0.763∗	0.692∗	0.013	1							
Mean Cortisol	-0.136	0.**252**∗∗	0.192	-0.152	**0.262**∗∗	1						
CAR	-0.136	0.150	-0.255	-0.067	-0.071	0.126	1					
AUC_G_	-0.107	**0.290**∗	0.161	-0.009	**0.311**∗	0.908∗	0.350∗	1				
DCS	-0.247	0.133	0.061	-0.410∗	0.018	0.611∗	-0.150	0.297∗	1			
Age	**-0.425**∗	0.080	-0.350	-0.315∗	**-0.371**∗	0.106	0.153	0.040	0.230	1		
Education	**-0.335**∗	0.315	-0.208	-0.0160	-0.408∗	-0.140	-0.131	-0.195	0.056	0.428∗	1	
Income	**-0.441**∗	0.458	-0.0244	-0.282∗	-0.571∗	-0.035	-0.050	-0.162	0.225	0.504∗	0.819∗	1
Stressor	**0.469**∗	0.190	-0.035	0.305∗	0.205	-0.162	-0.116	-0.198	-0.107	-0.337∗	-0.317∗	-0.392∗

Note: PM_2.5_ = Particulate Matter less than _2.5_ μm; CAR = Cortisol Awakening Response; AUC_G_ = Area Under the Curve; DCS = Diurnal Cortisol Slope. The bold values are representes the correlations of interest towards identifying trends.

∗ Correlation is significant at the 0.05 level (2-tailed).

∗∗ Correlation shows a trend toward significance at the 0.08 level (2-tailed).

The 3^rd^ trimester depression scores were used as the dependent variable in four time-specific PM_2.5_ exposure models (average PM_2.5_ exposure across pregnancy, exposure during the 1^st^ trimester, 2^nd^ trimester, and 3^rd^ trimester). Each time-specific model also included separate examination of each of the four different cortisol parameters (average cortisol, CAR, DCS, AUC_G_). Models were adjusted for maternal age, education, income, and exposure to prenatal stressors if preliminary bivariate relationships indicated they were significantly related to depression or the cortisol parameter being examined. The log likelihood-ratio test was applied in the analysis to identify the best fitting models. Analyses were conducted using Stata version 16 (StataCorp, College Station, TX).

## 3 Results

### 3.1 Sample characteristics

Our final sample size was 50 women who met the following criteria: recruited during their 3^rd^ trimester, had complete data for all variables needed to test study aims, and resided within 6 miles of a BAAQMD's monitor throughout their entire prenatal period. Sample characteristics and distributions for study outcome and exposure variables are displayed in Table 1. A distinct feature of our participants is their socioeconomic diversity. Women had an average age of 33 years. Approximately half of mothers received some college education or less (42%) with 22% having a high school education or less. 44% of mothers reported having an annual household income of $40,000 or less (32% reported $21,000 or less) while 50% had a household income of over $101,000. About 48% of the women were White/European American, 32% were Black/African American, 14% were of Hispanic/Latina ethnicity, and 6% were Asian American. 62% were married or lived with a partner while 24% were separated and 14% were single. Women reported having experienced from 0 to 39 life stressors over the previous 6 months, with an average of 7.3 stressful events.

The average depression score was 6.4, with values ranging between 1-20; however, log transformed values were used in the analysis. 80% of the women had minimal or mild symptoms of depression. 10% had moderate depression and 10% fell into the moderately severe to severe categories based on their PHQ-9 scores. Average prenatal PM_2.5_ exposure was 8.1 μg/m^3^, with exposure ranging from a mean of 7.4–8.8 μg/m^3^across various trimesters.

### 3.2 Bivariate correlations between key study variables

Table 2 presents the bivariate correlations for all PM_2.5_, depression, and cortisol variables as well as covariates. Among the particulate matter exposure variables, first trimester and average prenatal exposure to PM_2.5_ were most strongly correlated to a greater depression score (.296, p = .04; .343, p = .02; respectively). All covariates were strongly correlated to higher reported depression, with scores for the number of stressors experienced having the strongest correlation and greatest statistical significance (.469, p = .0006) followed by income (-.441, p = .0014), age (-.425, p = .0021), and education (-.335, p = .0174). No cortisol measure was significantly correlated with depression; however, first trimester and average prenatal exposure to PM_2.5_ were significantly correlated to the AUC_G_ cortisol measure (.290, p = .04; .311, p = .03; respectively) and showed trends toward significance in their correlation with women's average cortisol (.252, p = .08; .262, p = .07, respectively).

### 3.3 Effects of PM_2.5_ exposure on women's depressive symptoms during the 3^rd^ trimester (Aim 1)

Second and third trimester exposure to PM_2.5_ had no association to 3^rd^ trimester depressive symptoms (β = 0.036, p < 0.293; β = 0.056, p < 0.456, respectively). However, both PM_2.5_ exposure during the 1^st^ trimester and average PM_2.5_ exposure across pregnancy were associated with depression during the 3^rd^ trimester. Table 3 shows results from the linear regression models for 1^st^ trimester exposure (controlling for maternal age) and PM_2.5_ average pregnancy exposure (controlling for stressors incurred by women over the last 6 months). Income and education were included in preliminary model testing and found not to be significant, potentially due to their correlation with air pollution exposure, age and stressors which all had more robust effects on depression in final models and yielded better goodness of fit based on the likelihood-ratio test. Age and stressors were retained in the final models for testing effects of 1^st^ trimester PM^2.5^and average pregnancy PM^2.5^ exposures because they were the only covariates showing significance in their respective models. PM_2.5_ exposure significantly predicted depression in both models. According to our findings, per every 1 ug/m3 increase of PM2.5 exposure during both the first trimester and averaged across pregnancy, third trimester depression scores increased by 0.054 log units (p < 0.044) and 0.116 log units (p < 0.046), respectively. These changes would represent an increase in depressive symptoms of approximately 5% and 11% for every 1 ug/m3 increase of PM2.5 exposure during the first trimester and across pregnancy, respectively.

**Table 3:** Linear regression Models^a^ for the effects of both 1st trimester and average pregnancy PM2.5 exposures on 3rd trimester depressive symptoms.

Variable	β (95% CI)	Standard Error	p value	Variable	β (95% CI)	Standard Error	p value
1st trimester exposure	0.054 (0.002–0.106)	0.026	0.044	Average PM2.5 exposure	0.116 (0.002–0.230)	0.057	0.046
Age	-0.060 (-0.098 --0.022)	0.019	0.003	Stressors	0.045 (0.017–0.072)	0.013	0.002

a Log likelihood-ratio test was applied to identify the best fitting models. Depression scores were logarithmically transformed.

### 3.4 Mediating effects of cortisol in the relationship of PM_2.5_ exposure to women's depressive symptoms (Aim 2)

Eight structural equation models were computed to examine the mediating effects of the 4 cortisol parameters on average pregnancy PM_2.5_ exposure and 1^st^ trimester PM_2.5_ exposure. Two of the cortisol parameters showed the potential for a mediating effect in that PM_2.5_ exposure was significantly associated with these parameters: women's average cortisol level and their AUC_G_. These effects were present for PM_2.5_ exposure across pregnancy as well as for PM_2.5_ exposure during the 1^st^ trimester. Table 4 (average cortisol level) and 5 (AUC_G_) present results of the mediation models for effects of PM_2.5_ exposure across pregnancy on depression. Table 6 (average cortisol level) and 7 (AUC_G_) present results of the mediation models for effects of PM_2.5_ exposure during the 1^st^ trimester on depression.

**Table 4:** Structural equation model for the effects of average PM_2.5_ exposure across pregnancy on depressive symptoms as mediated by average cortisol level.

Direct Effects			Indirect Effects		Total Effects
Variables	Coef (95% CI)	p value	Coef (95% CI)	p value	Coef (95% CI) p value
**Effects on Average Cortisol**					
Average PM_2.5_ exposure	0.018 (0.003–0.034)	0.025	NP		0.018 (0.003–0.034) 0.025
Stressors	-0.003 (-0.006 – -0.001)	0.104	NP		-0.003 (-0.006 – -0.001) 0.104

**Effects on Depressive Symptoms**					
Average cortisol	-1.19 (-3.010 – 0.691)	0.206	NP		-1.19 (-3.010 – 0.691) 0.206
Average PM_2.5_ exposure	0.138 (0.025–0.248)	0.015	-0.021 (-0.060-0.017)	0.271	0.116 (0.008–0.224) 0.035
Stressors	0.041 (0.015–0.067)	0.002	0.003 (-0.004-0.011)	0.318	0.045 (0.019–0.070) 0.001

∗NP indicates No Path. Depression scores were logarithmically transformed.

Average PM_2.5_ exposure across pregnancy was significantly associated with both average cortisol level of women (0.018, p = 0.025; Table 4) and their cortisol AUC_G_ (15.93, p = 0.005; Table 5). Regardless, these cortisol parameters did not mediate the relationship between women's average pregnancy exposure to PM_2.5_ and their depressive symptoms. As shown in Table 4, average cortisol during pregnancy had no direct effect on depressive symptoms (-1.19, p = 0.206) nor was there any indirect effect of PM_2.5_ exposure on depressive symptoms that was mediated by average cortisol during pregnancy (-0.021, p = 0.27). Table 5 presents similar results for mediating effects of AUC_G_. AUC_G_ had no direct effect on depressive symptoms (-0.001, p = 0.331) nor was there any indirect effect of PM_2.5_ exposure on depressive symptoms that was mediated by AUC_G_ (-0.021, p = 0.359).

**Table 5:** Structural equation model for the effects of average PM_2.5_ exposure across pregnancy on depressive symptoms as mediated by cortisol AUC_G_.

Model Covariates	Direct Effect		Indirect Effect		Total Effect	
	Coef (95% CI)	p value	Coef (95% CI)	p value	Coef (95% CI)	p value
**Effects on AUC**_**G**_**Cortisol**						
Average PM_2.5_ exposure	15.933 (4.720–27.147)	0.005	NP		15.933 (4.720–27.147)	0.005
Stressors	-2.812 (-5.481 – -0.144)	0.039	NP		-2.812 (-5.481 – -0.144)	

**Effects on Depressive Symptoms**						
Cortisol AUC_G_	-0.001 (-0.004 – 0.001)	0.331	NP		-0.001 (-0.004 – 0.001)	0.331
Average PM_2.5_ exposure	0.137 (0.022–0.251)	0.019	-0.022 (-0.065-0.024)	0.359	0.116 (0.008–0.224)	0.035
Stressors	0.041 (0.014–0.067)	0.002	0.004 (-0.005-0.019)	0.379	0.045 (0.019–0.070)	0.001

NP indicates No Path. Depression scores were logarithmically transformed.

The models for PM_2.5_ exposure during the 1st trimester showed the same pattern of results as those for average PM_2.5_ exposure across pregnancy, with the exception that age as a covariate was included in the best fit models (Tables 6 and 7). First trimester PM_2.5_ exposure was significantly associated with both average cortisol level of women (0.038, p = 0.027; Table 6) and their cortisol AUC_G_ (6.737, p = 0.010; Table 7). Again however, these cortisol parameters did not mediate the relationship between women's 1^st^ trimester exposure to PM_2.5_ and their depressive symptoms, nor did they have a direct or indirect effect on depressive symptoms.

**Table 6:** Structural equation model for the effects of first trimester PM_2.5_ exposure on depressive symptoms as mediated by average cortisol level.

Direct Effect			Indirect Effect		Total Effect
Model covariates	Coef (95% CI)	P-value	Coef (95%)	P-value	Coef (95%) P-value
**Average Cortisol Effects**					
1^st^ trimester PM2.5	0.038 (0.004–0.072)	0.027	NP		0.038 (0.004–0.072) 0.027
Stressors	-0.022 (-0.040- -0.003)	0.023	NP		-0.022 (-0.040- -0.003) 0.023
Age	-0.010 (-0.016 – 0.035)	0.455	NP		0.010 (-0.016 – 0.035) 0.455

**Depressive Symptoms Effects**					
Average cortisol	-0.223 (-0.604 - 0.158)	0.252	NP		-0.223 (-0.604 - 0.158) 0.252
1^st^ trimester PM2.5	0.050 (0.003–0.010)	0.038	-0.008 (-0.025-0.008)	.309	0.043 (-0.004 - 0.090) 0.074
Stressors	0.030 (0.003–0.057)	0.027	0.005 (-0.004-0.014)	.306	0.035 (0.009–0.061) 0.008
Age	-0.042 (-0.078 - 0.007)	0.019	0.002 (-0.009-0.005)	.531	-0.044 (-0.080 - -.009) 0.015

NP indicates No Path. Depression scores were logarithmically transformed.

**Table 7:** Structural equation model for the effects of first trimester PM_2.5_ exposure on depressive symptoms as mediated by cortisol AUC_G_.

Direct Effect			Indirect Effect		Total Effect
Model covariates	Coef (95% CI)	P-value	Coef (95%)	P-value	Coef (95%) P-value
**AUC**_**G**_**Cortisol Effects**					
1^st^ trimester PM2.5	6.737 (1.579–11.896)	0.010	NP		6.737 (1.579–11.896) 0.010
Stressors	-2.787 (-5.633-0.059)	0.055	NP		-2.787 (-5.633-0.059) 0.055
Age	-.341 (-4.2370 – 3.555)	0.864	NP		-0.341 (-4.2370 – 3.555) 0.864

**Depressive Symptoms Effects**					
AUC_G_ Cortisol	-0.001 (-0.004 - 0.001)	0.387	NP		-0.001 (-0.004 - 0.001) 0.387
1^st^ trimester PM2.5	0.050 (0.001–0.010)	0.047	-0.007 (0.025–0.010)	0.413	0.043 (-0.004 - 0.090) 0.074
Stressors	0.032 (0.005–0.059)	0.018	0.003 (-0.005-0.001)	0.431	0.035 (0.009–0.061) 0.008
Age	-0.044 (-0.080 - -0.009)	0.013	0.000 (-0.004-0.005)	0.866	-0.044 (-0.080 - -0.009) 0.015

NP indicates No Path. Depression scores were logarithmically transformed.

## 4 Discussion

Based on the mean exposure of 8.1 μg/m^3^ found for our sample, most women experienced PM_2.5_ exposure levels below the standardized safety limit set by the EPA. However, the range of exposure among women went as high as 21 μg/m^3^ during their 1^st^ and 2^nd^ trimesters, suggesting exposure for some women beyond the established standard of less than or equal to 12.0 μg/m^3^ (National Ambient Air Quality Standards, 2020). In addition, the majority of women reported only minimal or mild symptoms of depression, although others incurred more severe depressive symptoms. Testing of Aim 1 indicated that women exposed to higher average levels of PM_2.5_ across pregnancy and, in particular, the 1^st^ trimester of their pregnancy, experienced greater depressive symptoms in their 3^rd^ trimester. There was no relationship between exposure to air pollution during the 2^nd^ and 3^rd^ trimesters and severity of depressive symptoms. Results for Aim 2 did not provide evidence of a mediating effect for any cortisol parameter in the association between exposure to particulate matter and depressive symptoms. However, elevated exposure to particulate matter was linked to greater cortisol concentration of women, evidenced in both higher average cortisol levels and their amount of overall cortisol secretion across the day.

### 4.1 PM_2.5_ exposure and depressive symptoms

Our findings regarding the lack of any effect for 2^nd^ and 3^rd^ trimester pollution are congruent with results from Lin et al. (2017) who found that exposure to PM10 during mid to late pregnancy was not associated with women's depression during pregnancy. However, we did find a relationship with exposure during the 1^st^ trimester which Lin and colleagues did not evaluate. The salience of the 1^st^ trimester may stem from the rapid and intense hormonal changes occurring at this point in pregnancy, especially increases in estrogen levels which soar during the first 12 weeks (Wharton et al., 2012). Research suggests that PM_2.5_ exposure can disrupt reproductive hormone levels (Wang et al., 2021) and induce hypermethylation in the estrogen receptor promoter region of the uterus (Dang et al., 2018). Such changes could have significant central nervous system implications for development of depression which has shown a robust relationship to estrogen levels (Albert et al., 2019). Additional studies indicate that the 1^st^ trimester may be a time of enhanced vulnerability to air pollution exposure, even at lower levels of pollution (Cai et al., 2017; Maghbooli et al., 2018). However, pollution exposure has been associated with changes in DNA methylation across the life course, with most research indicating a decrease in methylation throughout the genome (Rider and Carlsten, 2019). Studies are needed to clarify unique pollution effects on DNA methylation and its relationship to both depression and HPA axis dysregulation during pregnancy.

We found that elevated 1st trimester exposure to particulate matter and cumulative prenatal exposure across pregnancy predicted more severe depressive symptoms in the 3rd trimester. These findings suggest that effects of pollution on the Central Nervous System may take time to develop. This hypothesis is consistent with a recent meta-analysis reporting that long term exposure (>6 months) to PM_2.5_ was associated with a 10% increased risk of developing depression while findings for short term exposure were inconsistent in general populations (35). The link between long term exposure to pregnancy PM_2.5_ and depression has been observed in two previous studies. Sheffield et al. (2018) found that mid pregnancy exposure was related to depressive symptoms at 6 and 12 months postpartum, and Niedzwiecki et al. (2020) reported that average pregnancy exposure to PM_2.5_ was associated with the actual onset of postpartum depression at 6 months. However, neither study adjusted for trimester-specific depressive symptoms. Future studies should examine the trajectory of air pollution exposure across all trimesters of pregnancy and concurrent pollution-induced prenatal depressive symptoms as well as their influence on development of postpartum depression.

There is growing support for the relationship between PM_2.5_ exposure and development of depressive symptoms within varied populations, including women (Kioumourtzoglou et al., 2017), general adult populations (Braithwaite et al., 2019; Wang et al., 2018), individuals with chronic illness (Cho et al., 2014; Wang and Yang, 2018), children (Roberts et al., 2019), and older adults (Pun et al., 2017; Wang et al., 2014). In addition to the effects on DNA methylation noted above, other mechanisms have been proposed for this relationship, including increased oxidative stress, neuroinflammation, cerebrovascular damage, neurodegeneration, and dysregulation of the HPA axis (e.g. Block and Calderon-Garciduenas, 2009; Gladka et al., 2018; Tallon et al., 2017). Each of these mechanisms warrants exploration among pregnant women.

### 4.2 PM_2.5_ exposure and cortisol concentration

In our mediation analysis to test Aim 2, we did not find a mediating effect of any cortisol parameter, suggesting that cortisol dysregulation was likely not a potential mechanism in the relationship between PM_2.5_ and depressive symptoms. However, we did find that early pregnancy and average prenatal exposure to PM_2.5_ were associated with higher cortisol levels (women's average cortisol) and overall amount of cortisol women secreted throughout the day (AUCG). Both these findings indicate that higher levels of PM_2.5_ induced a greater concentration of cortisol in the women's system, implicating HPA axis activation as a result of exposure to particulate matter. This finding is supported by other research showing that exposure to particulate matter is significantly linked to higher levels of cortisol secretion (Li et al., 2017a, Li et al., 2017b; Niu et al., 2018), including during pregnancy (Khamirchi et al., 2020). Regardless of these effects, the cortisol parameters we examined did not, in turn, predict more severe depressive symptoms.

Although we know from previous research that hypercortisolism has been associated with development of depressive symptoms (Carroll et al., 2007; Nandam et al., 2020), our inclusion of other variables in our models (i.e. PM_2.5_ exposure, stressors and age), may have accounted for some of the variance that could potentially have been contributed by cortisol. Additionally, we found an inverse association between reported number of stressors and cortisol levels. Prior research has shown that burnout from stressors at work, exposure to chronic stressors and greater adversity can increase negative feedback inhibition in the HPA axis and lower cortisol levels (Bunea et al., 2017; Lennartsson et al., 2015). Enhanced negative feedback reduces synthesis of cortisol, resulting in lowered amounts of cortisol throughout the body (Heim et al., 2000). Another potential explanation is that individuals with higher daily stressors may begin to habituate to the stressors they endure or develop more extensive coping mechanisms to manage them. This could ultimately lead to some desensitization of the HPA axis and lower cortisol levels (Rosal et al., 2004). However, further investigation is needed to examine these hypotheses.

**Figure 2:**
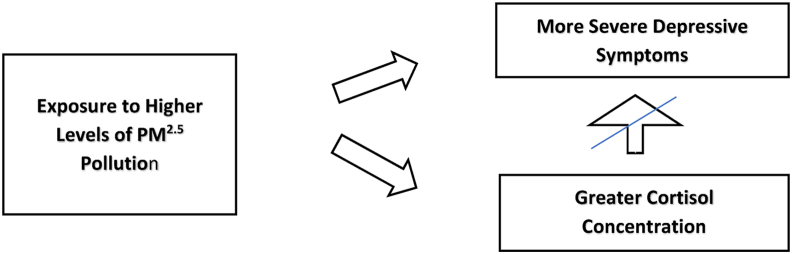
Air pollution independently predicts cortisol concentration and depressive symptoms.

Figure 2 shows the revised model emerging from our results, with higher levels of PM_2.5_ exposure during pregnancy independently contributing to greater cortisol concentration among women and more severe depressive symptoms, but with no mediating role for cortisol concentration.

### 4.3 Study strengths and limitations

Our study includes several strengths, including our diverse economic and racial sample, the high temporal resolution of the PM_2.5_ estimates, control for women's stressors and age where indicated in model testing, and our assessment of the mediating effects of cortisol in understanding the relationship between air pollution exposure and depression. However, there are also limitations. Our PM_2.5_ subject assigned exposure data was reliant on the nearest single site monitor from a sparse network of ground monitors. We also did not account for other high frequent PM_2.5_ exposures at sites such as the workplace or indoor air pollution levels that may have affected women's overall exposure. Further, we did not assess other air pollutants that may co-occur with PM_2.5_ and could potentially confound our associations. Our depression measure and our cortisol samples were acquired in the 3^rd^ trimester rather than enabling their concurrent assessment with pollution estimates over the entire pregnancy. While we found no association between cortisol and depression, the dysregulated HPA axis has shown robust effects on the development of depression in previous research. Our limited sample size and the cross-sectional nature of our study may have reduced our ability to detect significant effects.

## 5 Conclusions

Our findings indicate that air pollution may be a modifiable risk factor for cortisol dysregulation and for depression during pregnancy. Both regulatory policies and interventions to reduce pollution exposure could decrease women's vulnerability to elevated stress hormones and depression during pregnancy. Such interventions have implications for preventing unnecessary suffering of women as well as decreasing adverse birth outcomes associated with both depression and cortisol dysregulation during pregnancy. Our results add to the growing demand for policy changes to reduce pollution exposure and mitigate the increasing prevalence of prenatal depression. In addition, they point to the need for assessment of depression early in pregnancy in regions known for high pollution to prevent further exacerbation of adverse perinatal mood effects. In conjunction with policy changes related to addressing elevated neighborhood air pollution levels, clinical efforts can assist with the modification of increased air pollution exposure at the individual level. Prenatal screening for pollution could enhance more precise identification of risk for depression that is not evident yet in depression measures and lead to practical recommendations to mitigate depression (e.g. air purifiers). Although our results suggest that cortisol does not mediate the effects of pollution on depression, future research should include a larger sample as well as concurrent measures of depression, cortisol and pollution across pregnancy and the postpartum to better understand the trajectory of their inter-related effects and their potential interactions.

## Declarations

### Author contribution statement

Nina Ahlers: Conceived and designed the experiments; Performed the experiments; Analyzed and interpreted the data; Contributed reagents, materials, analysis tools or data; Wrote the paper.

Sandra Weiss: Conceived and designed the experiments; Analyzed and interpreted the data; Contributed reagents, materials, analysis tools or data; Wrote the paper.

### Funding statement

This study was funded by a grant from 10.13039/100000052NIH (NICHD RO1 HD081188-05) to S. Weiss, PI.

### Data availability statement

Air pollution data extracted from the Bay Area Air Quality Monitoring District.

### Declaration of interests statement

The authors declare no conflict of interest.

### Additional information

No additional information is available for this paper.
